# Analysis of gene expression of secreted factors associated with breast cancer metastases in breast cancer subtypes

**DOI:** 10.1038/srep12133

**Published:** 2015-07-15

**Authors:** Elana J. Fertig, Esak Lee, Niranjan B. Pandey, Aleksander S. Popel

**Affiliations:** 1 Department of Oncology, Sidney Kimmel Comprehensive Cancer Center, Johns Hopkins University, Baltimore, MD, USA; 2 Department of Biomedical Engineering, Johns Hopkins University School of Medicine, Baltimore, MD, USA

## Abstract

Breast cancer is a heterogeneous disease, having multiple subtypes with different malignant phenotypes. The triple-negative breast cancer, or basal breast cancer, is highly aggressive, metastatic, and difficult to treat. Previously, we identified that key molecules (IL6, CSF2, CCL5, VEGFA, and VEGFC) secreted by tumor cells and stromal cells in basal breast cancer can promote metastasis. It remains to assess whether these molecules function similarly in other subtypes of breast cancer. Here, we characterize the relative gene expression of the five secreted molecules and their associated receptors (GP130, GMRA, GMRB, CCR5, VEGFR2, NRP1, VEGFR3, NRP2) in the basal, HER2 (human epidermal growth factor receptor 2) positive, luminal A, and luminal B subtypes using high throughput data from tumor samples in The Cancer Genome Atlas (TCGA) and Molecular Taxonomy of Breast Cancer International Consortium (METABRIC). *IL6* and *CCL5* gene expression are basal breast cancer specific, whereas high gene expression of *GP130* was observed in luminal A/B. *VEGFA/C* and *CSF2* mRNA are overexpressed in HER2 positive breast cancer, with *VEGFA* and *CSF2* also overexpressed in basal breast cancer. Further study of the specific protein function of these factors within their associated cancer subtypes may yield personalized biomarkers and treatment modalities.

Breast cancer is the most frequently diagnosed cancer among women in the United States^1^. Primary breast tumors are divided in four main molecular subtypes: Basal (also known as, triple negative), HER2 (human epidermal growth factor receptor 2) positive, Luminal A, and Luminal B. Each of these subtypes has characteristic traits and expected patient outcome. For example, basal breast cancer is the most aggressive and metastatic subtype. Basal breast tumors do not express typical breast cancer cell receptors, such as the estrogen receptor (ER), the progesterone receptor (PR), and does not overexpress the human epidermal growth factor receptor 2 (HER2) that are activated in the other subtypes^2^. Thus, current hormonal therapies and HER2 inhibition cannot be used to treat basal breast cancer. Moreover, therapeutic resistance is common when treating tumors from other subtypes with hormonal therapies^3^. Therefore, new therapeutics that target additional molecular factors in breast tumors are needed. Optimal therapeutics would target the factors that promote tumor growth and metastasis resulting from interactions between cancer cells, stromal cells, and extracellular matrix.

Secreted factors from each of the diverse cells in a tumor regulate inter-cellular signaling between tumor cells and the microenvironment to promote breast cancer growth and metastasis^4^. Specifically, the secreted factors cytokines, chemokines, and growth factors contribute to distinct modes of metastasis and subsequent mortality^5^. Cytokines represent soluble proteins secreted by mammalian cells that are important in cell signaling. Among them, cytokines related to inflammatory signals are involved in many human diseases. For example, in cancer, inflammation induces tumor growth, tumor drug resistance, and metastasis^6^. Chemokines have been studied in immunology and are known to serve as immune cell-recruiting and cell-trafficking factors^7^. Though chemokines are a subset of cytokines, they are categorized as distinct secreted factors for this study, as they can play a role in tumor cell motility and recruitment, which are critical for metastatic dissemination. Growth factors are essential secreted factors for cancer cell proliferation, maintenance, migration, and adhesion^8^. Angiogenic and lymphangiogenic growth factors can regulate angiogenesis and lymphangiogenesis in both primary tumors and pre-metastatic niches^9^,^10^.

Because of their critical role activating the signaling processes responsible for tumor maintance and progression, tumor secreted factors (“tumor secretome”) can serve as targets to inhibit the primary tumor growth^11^. The specific secreted factor in an individual tumor would provide the most promising therapeutic target in that individual’s disease. Therapeutics that thus target the secretome have the greatest potential for translation to the clinic when the factor they target are common to multiple tumors from each molecular subtype.

We previously reported that the secreted factors IL6, CSF2, CCL5, VEGFA, and VEGFC are pivotal orchestrators of basal breast cancer growth and metastasis^4^,^12^. Specifically, this previous study reported that basal breast cancer cells secrete interleukin 6 (IL6), a cytokine, which conditions (educates, reprograms) lymphatic endothelial cells (LEC) within pre-metastatic organs and primary tumors to secrete the chemokine CC-chemokine ligand 5 (CCL5) and the growth factor, vascular endothelial growth factor A (VEGFA)^4^. This group of secreted factors including a cytokine, a chemokine, and a growth factor make a self-reinforcing paracrine loop to promote basal breast cancer metastasis. LEC-derived CCL5 recruits CCR5-positive cancer cells into the lymphatic vessels and triggers tumor dissemination. LEC-derived VEGFA interacts with blood endothelial cells (BEC) of the vasculature enhancing vascular permeability in the lungs, and promoting angiogenesis in the lymph nodes. These are important steps for tumor cell extravasation and colonization. VEGFC is a lymphangiogenic growth factor, secreted by cancer cells and stromal cells to promote lymphatic vessel growth in primary tumors^13^. We also demonstrated a crosstalk between basal breast cancer cells and BEC/LEC that was important for primary tumor growth^12^. We showed that the secretomes of the BEC and LEC are perturbed in distinct ways, influencing primary tumor growth, pericyte infiltration, and angiogenesis in different ways^12^.

In addition to having a pivotal role in basal breast cancer growth and metastasis, the secreted factors implicated in our previous study (IL6, CSF2, CCL5, VEGFA, and VEGFC) may also serve critical roles in other subtypes of breast cancers. In this case, inhibitors of these factors and the pathways they regulate could be used to treat a wider array of breast cancers. High throughput genomic data can indicate the molecular profile of these factors to infer such candidate targets from the secretome. Therefore, in this study we identify the relative gene expression of IL6, CSF2, CCL5, VEGFA, and VEGFC and their receptors (GP130, GMRA, GMRB, CCR5, VEGFR2, NRP1, VEGFR3, and NRP2, Fig. 1), in multiple breast cancer subtypes (Basal, HER2+, Luminal A, and Luminal B) using high-throughput genomic data of primary tumors from the Cancer Genome Atlas (TCGA)^14^ and Molecular Taxonomy of Breast Cancer International Consortium METABRIC^15^. The goals of this study are: (a) to understand how these key pro-metastatic factor genes are expressed in breast cancer subtypes, (b) to examine how their associated receptor genes are expressed in the subtypes, and (c) to evaluate whether these gene expression profiles can predict the survival rates within each breast cancer subtype.

## Results

### Selection of secreted factors associated with metastasis

**Figure 1:**
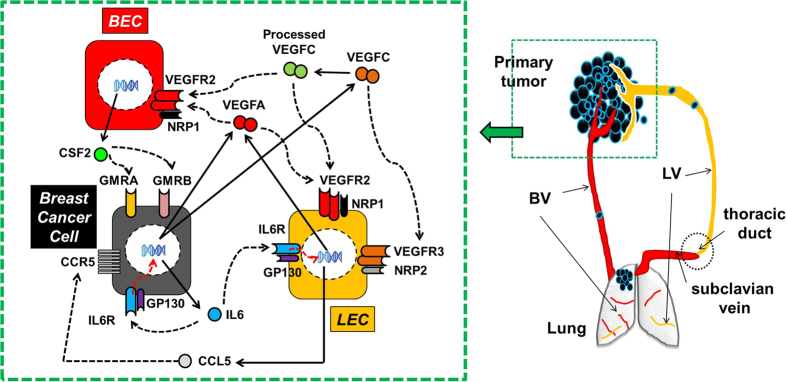
Summary of relationship of ligand and receptor pairs previously associated with metastasis (Lee *et al.* 2014) in tumor cells and adjacent BEC and LEC.

Figure 1 shows the ligand and receptor interactions between the factors we previously associated with metastasis^4^ and have selected for analysis in this current study (listed in Table 1). Specifically, this figure summarizes the relationship between these factors and adjacent blood endothelial cells (BEC) and lymphatic endothelial cells (LEC) from our previous study. Primary tumor samples used for genomic profiling contain a mixture of tumor cells and cells from the microenvironment, including adjacent BEC and LEC. We test whether the putative regulatory relationships between ligands and their associated receptor(s) are represented in mRNA expression of such primary tumors by correlating mRNA expression of each ligand with its associated receptor(s). Correlation analyses are run on large cohorts of gene expression data from 638 primary tumors in TCGA (Table 2) and from 897 primary tumors in METABRIC (Table 2), with sample characteristics in Supplemental Tables 1 and 2, respectively.

In TCGA, *CSF2*, *CCL5*, and *VEGFC* are all significantly correlated to their target receptors (*GMRA* and *GMRB*; *CCR5*; and *VEGFR3* and *NRP2*, respectively, Table 2). *VEGFA* is significantly correlated to only one of its receptors (*VEGFR2*), but not to the other target receptor *NRP1*. *IL6* is significantly anti-correlated with its target receptor (*GP130*). The significant anti-correlation between *IL6* and *GP130* and correlation between *VEGFC* and *NRP2* and *CSF2* and targets *GMRA* and *GMRB* are all confirmed in gene expression data from METABRIC (Table 2). However, the correlation between *VEGFC* and target receptor *VEGFR3* fails to meet statistical significance. We are also unable to confirm associations with *CCL5* with *CCR5* or *VEGFA* with *VEGFR2* because the array used in METABRIC does not contain probes that measure gene expression of *CCR5* or *VEGFA*.

### IL6 is overexpressed in basal breast cancer while its receptor GP130 is overexpressed in luminal breast cancer

**Figure 2:**
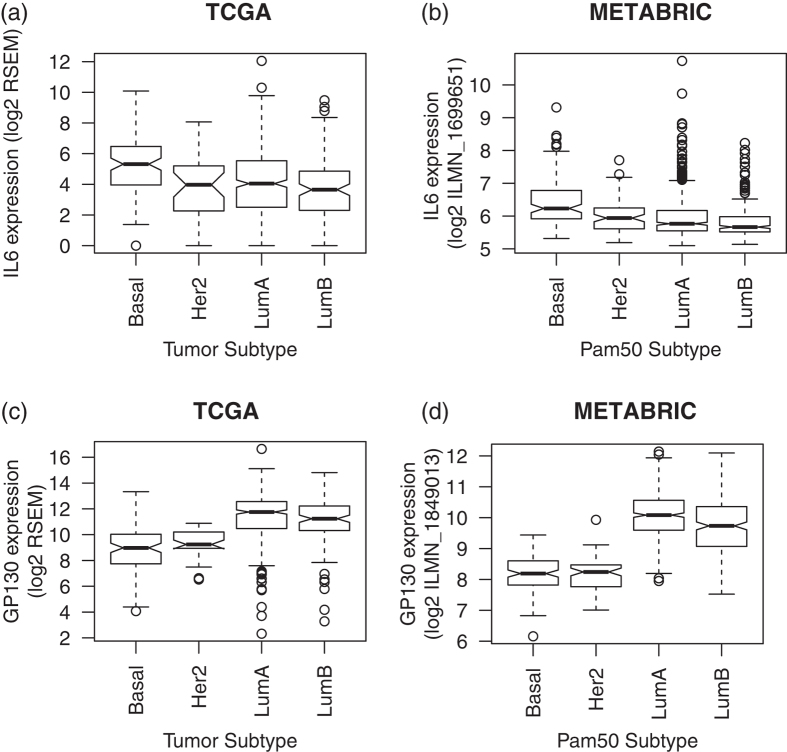
Gene expression of *IL6* in (**a**) TCGA by tumor subtype and in (**b**) METABRIC by Pam50 gene expression subtype. (**c**) and (**d**) provide corresponding boxplots for expression of the *IL6* ligand target receptor *GP130*.

We compared expression of the ligand *IL6* and its target receptor *GP130* in each of the breast cancer subtypes. *IL6* is significantly overexpressed in the basal subtype relative to other subtypes in both TCGA (p-value of 5 × 10^−8^) and METABRIC (p-value of 1 × 10^−9^) data (Fig. 2a,b, respectively). On the other hand, the target receptor *GP130* is significantly overexpressed in both luminal subtypes (Fig. 2c for TCGA and 2d for METABRIC with corresponding one-sided p-values below 2 × 10^−16^ in both datasets). Survival analyses were run for these genes using only METABRIC, due to the relatively long patient follow-up times in that dataset. We observed a trend towards longer survival times based upon *GP130* (Supplemental Fig. 3) expression in the luminal A subtype (p-value of 0.06) not observed in the other subtypes; no significant trend was observed for *IL6* (Supplemental Fig. 4).

### Overexpression of CSF2 target receptors GMRA and GMRB are associated with survival in basal and HER2+ breast cancer

**Figure 3:**
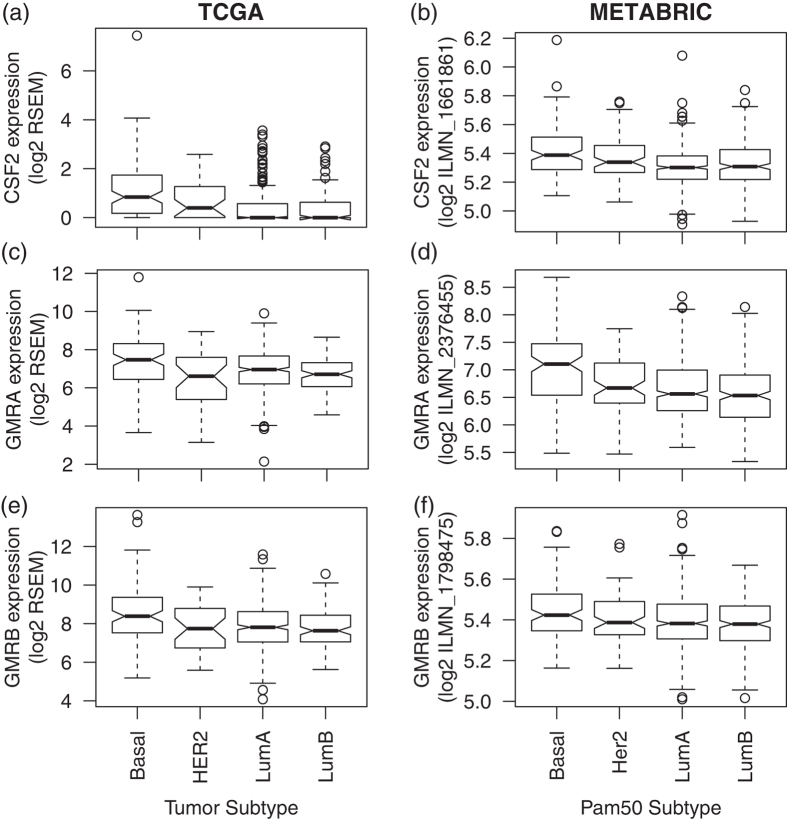
Gene expression of *CSF2* in (**a**) TCGA by tumor subtype and in (**b**) METABRIC by Pam50 gene expression subtype. (**c**) and (**d**) provide corresponding boxplots for expression of the *CSF2* ligand target receptor *GMRA* and (**e**) and (**f**) for *GMRB* in TCGA and METABRIC, respectively.

In TCGA, *CSF2* is significantly overexpressed in basal and HER2+ breast cancer relative to luminal subtypes (Fig. 3a, p-values of 1 × 10^−8^ and 0.03, respectively). This discrepancy in p-values is consistent with a higher log fold change in basal relative to luminal breast cancer (0.7) than HER2+ relative to luminal breast cancer (0.3). A similar trend is confirmed in METABRIC (Fig. 3b), with a p-value of 1 × 10^−8^ with log fold change of 0.1 in basal breast cancer relative to luminal and p-value 0.006 with log fold change of 0.05 in HER2+ relative to luminal. However, in both datasets target receptor *GMRA* was only overexpressed in basal breast cancer relative to all other subtypes (Fig. 3c,d with p-values of 2 × 10^−4^ for TCGA and 7 × 10^−10^ for METABRIC). Similar association with basal breast cancer was observed for the other target receptor, *GMRB* (Fig. 3e,f and p-values of 1 × 10^−4^ for TCGA and 9 × 10^−4^ for METABRIC).

*CSF2* expression was not significantly associated with survival in any subtypes (Supplemental Fig. 5). Nonetheless, higher *GMRA* expression significantly associated with better survival in basal breast cancer (Supplemental Fig. 6, p-value of 0.02) and *GMRB* expression trended towards higher survival in HER2+ breast cancer (Supplemental Figure 7, p-value of 0.08).

### CCL5 overexpression is associated with basal breast cancer and with survival in HER2+ breast cancer

**Figure 4:**
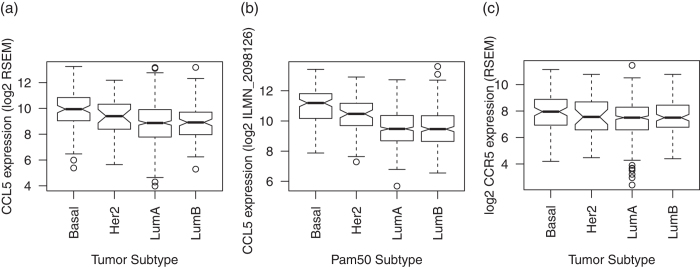
Gene expression of *CCL5* in (**a**) TCGA by tumor subtype and in (**b**) METABRIC by Pam50 gene expression subtype. Corresponding boxplot of expression of *CCL5* target receptor, *CCR5*, in TCGA is in (**c**).

Similar to *CSF2*, chemokine *CCL5* is significantly overexpressed in basal over luminal breast cancer in both TCGA (Fig. 4a, p-value of 9 × 10^−8^) and METABRIC (Fig. 4b, p-value below 2 × 10^−16^). *CCL5* is also significantly overexpressed in HER2+ breast cancer relative to luminal in METABRIC (p-value of 9 × 10^−9^) with a similar trend that failed to reach statistical significance in TCGA (p-value of 0.09). Likewise, its target receptor CCR5 is significantly overexpressed only in basal breast cancer in TCGA (Fig. 4c, p-value of 0.02). We are unable to confirm these relationships in METABRIC because there was no associated probe for this gene on the array measuring expression in this study. Nonetheless, increased *CCL5* expression was associated with better survival in HER2+ breast cancer (Supplemental Figure 8, p-value of 0.01).

### VEGFA is associated with HER2+ breast cancer and its target VEGFR2 with survival in basal breast cancer

**Figure 5:**
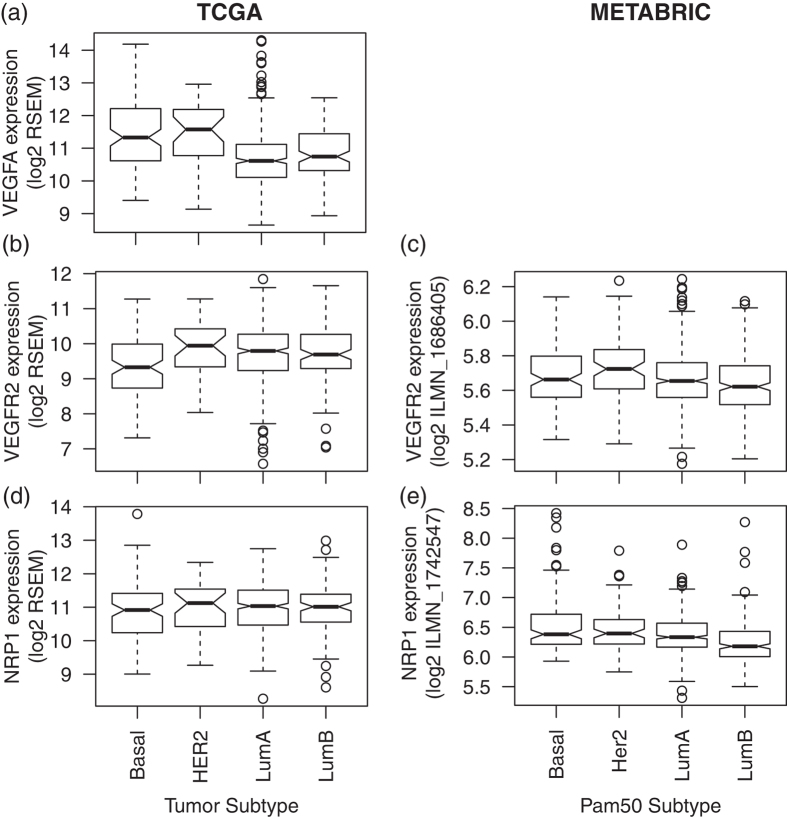
Gene expression of *VEGFA* in(**a**) TCGA by tumor subtype and in (**b**) METABRIC by Pam50 gene expression subtype. (**c**) and (**d**) provide corresponding boxplots for expression of the *VEGFA* ligand target receptor *VEGFR2* and (**e**) and (**f**) for *NRP1* in TCGA and METABRIC, respectively.

In TCGA data, *VEGFA* expression is highest in HER2+ breast cancer (Fig. 5a, p-value of 5 × 10^−3^ relative to other subtypes). It is also overexpressed in basal breast cancer relative to luminal breast cancer (p-value of 5 × 10^−9^). These relationships could not be confirmed in METABRIC because no probe measures *VEGFA* gene expression.

Although expression of the target receptor *VEGFR2* was not associated with any subtype in TCGA data (Fig. 5b), it was significantly overexpressed in HER2+ breast cancer in METABRIC (Fig. 5c, p-value of 4 × 10^−3^). The other target receptor, *NRP1*, was not differentially expressed in any breast cancer subtypes in TCGA (Fig. 5d), but was overexpressed in basal breast cancer relative to other subtypes in METABRIC (Fig. 5e, p-value of 2 × 10^−4^). Moreover, increased *VEGFR2* expression was significantly associated with better survival in basal breast cancer (Supplemental Figure 9, p-value of 0.008). No significant associations with survival were observed in any subtype for *NRP1* (Supplemental Figure 10).

### VEGFC is significantly overexpressed in HER2+ breast cancer

**Figure 6:**
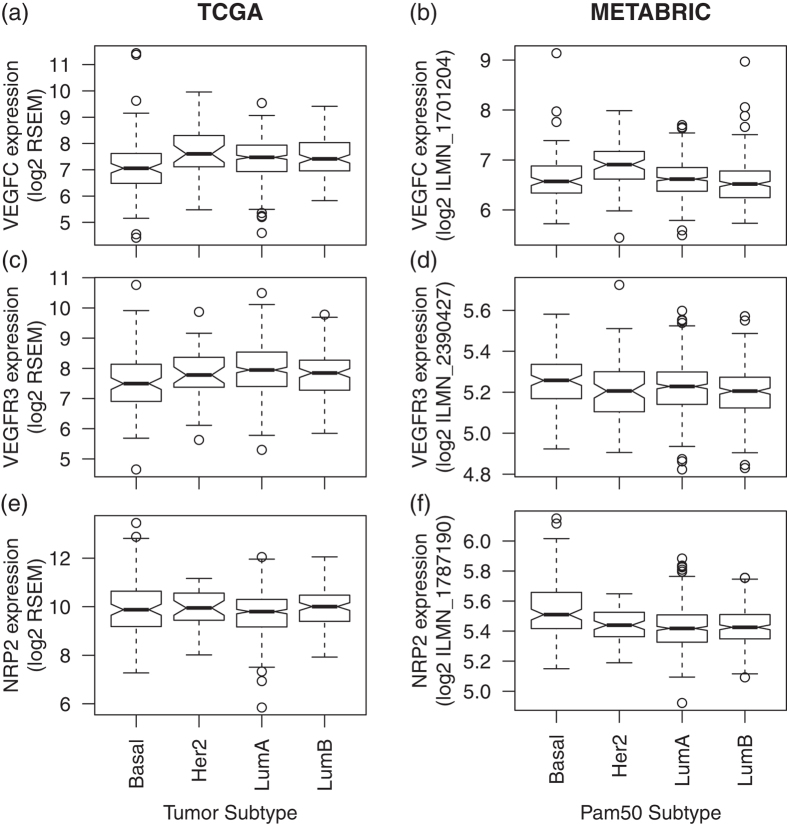
Gene expression of *VEGFC* in (**a**) TCGA by tumor subtype and in (**b**) METABRIC by Pam50 gene expression subtype. (**c**) and (**d**) provide corresponding boxplots for expression of the *VEGFC* ligand target receptor *VEGFR3* and (**e**) and (**f**) for *NRP2* in TCGA and METABRIC, respectively.

*VEGFC* is significantly overexpressed in HER2+ breast cancer relative to basal breast cancer in TCGA (Fig. 6a, p-value of 0.005) and in HER2 + breast cancer relative to all other subtypes in METABRIC data (Fig. 6b, p-value of 4 × 10^−6^). It is also overexpressed in both luminal subtypes relative to basal breast cancer in TCGA (p-value of 0.003), not confirmed in the METABRIC data. We observe significant overexpression of target receptor *VEGFR3* in the luminal A subtype in TCGA (Fig. 6c, p-value of 2 × 10^−3^), while we observe significant overexpression of *VEGFR3* in basal breast cancer in METABRIC (Fig. 6d, p-value of 9 × 10^−3^). *NRP2*, which is not significantly differentially expressed in any subtype in TCGA (Fig. 6e) but is significantly overexpressed in basal breast cancer in METABRIC (Fig. 6f, p-value of 9 × 10^−8^). Moreover, we do not observe any significant survival differences based upon expression of genes in this pathway (Supplemental Figures 11–13 for *VEGFC*, *VEGFR3*, and *NRP2*, respectively).

## Discussion

In this study, we characterized gene expression of multiple secreted factors and their receptors, in all the breast cancer subtypes (Basal, Her2, Luminal A, and Luminal B) by using large genomic studies (TCGA and METABRIC). The factors and receptors analyzed were *IL6, CSF2, CCL5, VEGFA, VEGFC, GP130, GMRA, GMRB, CCR5, VEGFR2, NRP1, VEGFR3, and NRP2*, because we found their protein expression to be critical to basal breast cancer metastasis in previous studies^4^,^12^. In this study, we found that that *IL6* and *CCL5* gene expression are basal breast cancer specific. High gene expression of *GP130* is observed in luminal A/B. *VEGFA/C* and *CSF2* mRNA are overexpressed both basal and HER2+ breast cancer relative to luminal subtypes.

*IL6* mRNA expression is higher in basal breast cancer when compared to other subtypes of breast cancer. Basal breast cancer is considered as aggressive and metastatic, and effective therapeutic treatments are very limited. IL6 protein has been studied in breast cancer^16^; it promotes formation of cancer stem cells^17^. Mesenchymal stem cell derived IL6 protein promotes breast cancer cell migration and invasion^18^. IL6 protein is also involved in drug resistance in breast cancer^19^. Studies of protein expression in basal breast tumors during lymph node metastasis in mouse models have shown that IL6 was highly expressed in lymph node positive basal breast tumors, compared to lymph node negative basal breast^20^. IL6 protein can activate a STAT3 (signal transducer and activator of transcription 3) pathway. Binding of IL6 protein to the GP130 receptor triggers STAT3 phosphorylation by JAK2^21^. Recent bioinformatics study showed that STAT3-associated genes can be a prognostic marker in basal breast cancer^22^. Although we did not observe an association between *IL6* gene expression and survival, the association of *IL6* gene expression with basal breast cancer and its protein function documented in other previous studies suggest that IL6 protein expression may serve as a therapeutic and diagnostic marker for basal breast cancer growth and metastasis.

Surprisingly, we observed that *GP130*, a functional receptor gene for IL6 signal transduction, has lower mRNA expression in basal breast cancer, compared to luminal breast cancer. At the same time, *IL6* mRNA was not highly expressed in luminal breast cancer, but enriched in basal subtype. This was unexpected because autocrine signaling of IL6-GP130-STAT3 in basal cancer cells is well-studied^23^,^24^,^25^. This discrepancy may be attributed to differences between gene expression and protein expression or function. Specifically, the membrane receptor *GP130* mRNA must be translated into protein and then bind IL6 protein to function as a signal transducer. Thus, functional studies at the protein level are needed. Nonetheless, lower *GP130* gene expression is consistent with reduced GP130 protein expression observed in a recent study by Lee *et al.*^26^. Specifically, this study showed that multiple cancer cells (e.g., lymphoma, adenocarcinoma, breast and prostate cancer) maintain activated STAT3 persistently via SIP-S1PR1 signaling, without IL6-GP130 signaling^26^. Sphingosine-1-phosphate receptor-1 (S1PR1), a receptor for the sphingosine-1-phosphate (S1P), is elevated in STAT3-positive tumors. S1P-S1PR1-induced STAT3 activation is persistent, in contrast to transient STAT3 activation by IL6 protein. This may suggest that basal breast tumor with lower mRNA expression of *GP130* may employ other pathways, such as S1P-S1PR1^27^. Since paracrine roles of IL6 protein are relatively less understood, our data suggest that *IL6* mRNA expressed by basal tumor cells can play a role in paracrine activators for other types of breast cancer cells (e.g., luminal) or stromal cells. It has been reported that HOXB13 protein mediates tamoxifen resistance and invasiveness in luminal breast cancer by suppressing estrogen receptor (ER) and inducing IL6 protein expression^28^. This study demonstrates that IL6 signaling promotes aggressiveness in luminal breast cancer cells making them more basal-like. We also showed that IL6 protein expressed by basal cancer cells activated other stromal cells (e.g., lymphatic endothelial cells) to promote tumor metastasis. Paracrine roles of the IL6 protein are still less understood, warranting further studies, particularly in the luminal subtype of breast cancer and in other stromal cells in basal breast cancer.

In addition to *IL6*, *CCL5* mRNA expression is highest in basal breast cancer, which is consistent with our experimental study showing that IL6 protein expressed by basal cancer cells conditions LEC to overexpress CCL5 protein^4^. That previous study also reported a significant correlation between *CCL5* and *IL6* gene expression in lymph node positive basal breast cancer samples from TCGA; there was no correlation for lymph node negative samples. We note that our previous study associated CCL5 protein expression in the lungs and lymph nodes with metastatic potential, but not normal LECs or cancer cells. Whereas that study analyzed protein expression in isolated cell types, the present study analyzes gene expression in primary tumors. The primary tumor samples in this study are from not purified cancer cells. As a result, this study cannot quantify the expression of *CCL5* in distinct cell types. Therefore, further study is required to establish the relative expression of *CCL5* in distinct cell types or metastatic sites suggested in our previous study. Nonetheless, the primary samples contain a mixture of cells so that the gene expression profiling may also characterize expression from LECs that are located in the tumor and express CCL5. We therefore hypothesize that *IL6* and *CCL5* gene expression within basal cancer tumor samples may determine their metastatic potential.

In this study higher *CCL5* gene expression is associated with better prognosis in HER2+ breast cancer samples, but not basal breast cancer. The survival data are in contrast to association of CCL5 protein expression with metastatic potential in basal breast cancer. Nonetheless, the role of CCL5 in HER2+ breast cancer metastases warrants further study. For example, HER2+ breast cancer shows high rate of brain metastasis, compared to other subtypes^29^,^30^ and shows severe drug resistance^31^. Our previous study found that both of these phenotypes are consistent with CCL5 overexpression. Moreover, recently, a STAT3-CCL5 loop was studied in drug resistance in luminal cancer^19^ and distal metastasis in basal breast cancer^4^. As was the case for GP130, differences may be attributed to discrepancies between measurements of mRNA expression and protein function. Discrepancy between *CCL5* gene expression and survival in HER2+ breast cancer may also arise from confounding clinical factors in the survival analysis that are independent of either therapeutic resistance or metastatic site. Associations of CCL5 expression with time to metastasis or metastatic site may be more consistent with the metastatic potential established in previous studies. However, adequate clinical data for these analyses are not available for either METABRIC or TCGA. Future prospective studies are required to establish the link between CCL5 expression, metastatic potential, and survival in breast cancer subtypes. The CCL5 protein may alternatively play a different role in HER2+ breast cancer and must be studied in tumor-drug resistance.

VEGFA and VEGFC are angiogenic and lymphangiogenic growth factors, respectively. We showed that mRNA expression for these growth factors is highest in HER2+ breast cancer compared to other subtypes and that *VEGFR2* mRNA expression predicted survival in HER2 patients. It has been shown that HER2 and angiogenesis signaling pathways exhibit molecular crosstalk^32^. In that study, higher microvascular density (enhanced angiogenesis) in human breast tumor samples predicted higher co-expression of HER2 and ER^33^. Angiogenesis impairment in Id-deficient mice completely suppressed HER2/neu-dependent breast tumors^34^, suggesting a role of angiogenic and lymphangiogenic growth factors in supporting tumor growth and hematogenous metastasis. Nonetheless, higher mRNA expression is associated with better prognosis in HER2+ breast cancer, similar to inconsistences between the metastatic potential and survival of HER2+ breast cancer for *CCL5*. *NRP1* was overexpressed in basal breast cancer relative to other subtypes in METABRIC (Fig. 5e). It has been reported that expression of both VEGF and semaphorin genes are altered in basal breast cancer^35^. Semaphorin proteins are ligands of NRP proteins and exhibit anti-angiogenic and anti-lymphangiogenic property^36^. A pattern of high VEGFA expression with low expression of secreted semaphorins was associated with 60% of basal breast tumors^35^. Though *VEGFC* mRNA expression in HER2+ breast cancer is less well-understood, recent study showed that HER2/neu expression correlates with VEGFC and lymphangiogenesis in lymph node-positive breast cancer^37^. Molecular crosstalk between *VEGFC* mRNA expression and HER2 / HER2-dependent transcription factors remains to be investigated in future studies.

We also found that *GMRA and GMRB*, possible receptors for *CSF2 (GM-CSF)*, to have high gene expression in basal breast cancer. Their high mRNA expression in HER2+ breast cancer was correlated with better survival. Roles of GM-CSF signaling in breast cancer are still controversial. GM-CSF is known as either an anti-tumorigenic host immune booster^38^,^39^ or anti-angiogenic factor^40^ or pro-metastatic factor^41^. These suggest that targeting CSF2 signaling must be considered carefully, and needs to be further clarified with more mechanistic studies.

In summary, we analyzed expression of pro-metastatic factor genes in breast cancer subtypes and showed correlation between factor/receptor gene expression and patient survival rates using TCGA and METABRIC datasets. From the study, we found that *IL6* and *CCL5* are overexpressed in basal breast cancer, suggesting their potential as therapeutic targets. It remains to be determined if VEGFA and its receptor VEGFR2 and VEGFC and its receptor VEGFR3, and CSF2 and its receptor GMRA/GMRB can also serve as therapeutic targets for HER2+ breast cancer since the associations we found were modest. High levels of gene expression of *IL6* receptor, *GP130*, in luminal A and B warrant further studies of paracrine roles of IL6 in luminal cancer. Other cell types such as tumor-associated macrophages can contribute to secretion of IL6 in tumor microenvironment; this has been observed in other tumor types^42^,^43^,^44^. In our previous study, we showed by immunohistochemistry that CCL5 protein expression was co-localized with LECs in the lungs in tumor-bearing mice, however, normal mice without tumors did not show CCL5 expression in LEC^4^. However, the primary tumor samples profiled in both TCGA and METABRIC contain a mixture tumor cells and cells in the microenvironment. Therefore, future screening studies of the protein expression of these factors on microdissected tissues must be performed to assess regulatory relationships within the different subtypes for treatment selection. Future prospective studies of associating *CCL5, VEGFR2*, and *CSF2* expression with metastases could mitigate the confounding factors that may be contributing to contradictory associations in the survival analyses.

## Methods

### Primary breast cancer gene expression data in TCGA

Analyses of TCGA data^14^ are performed on primary breast cancer tumor samples with both RNA-sequencing data and clinical annotations. Level 3 normalized gene expression (RNA Seq V2) is obtained from cBioPortal using the CRAN CGDS-R package (version 1.1.30)^45^. Gene expression data is log2 transformed and subset to the genes of interest in Fig. 1. The following aliases identified from the Bioconductor package org.Hs.eg.db (version 2.14.0) are used to match the gene annotations used in the TCGA alignment and normalization pipeline according to Table 2.

Clinical data for each TCGA sample is downloaded directly from the TCGA Data Portal. ER and PR status are assessed using the consensus of clinical tests and summarized in “breast carcinoma estrogen receptor status” and “breast carcinoma progesterone receptor status”, respectively. HER2 status is obtained from IHC in the variable labeled “lab proc her2 neu immunohistochemistry receptor status.” Samples missing data for any one of these tests are excluded from analysis, leaving a total of 638 samples with RNA-sequencing data. Breast cancer samples are defined as “Basal” if all three markers are negative, “HER2+” if only HER2 is positive, “Luminal A” if either ER or PR are positive but not HER2, and “Luminal B” if HER2 is positive in addition to either ER or PR. Supplementary Table 1 summarizes the clinical attributes of each of the 638 samples by these subtypes.

### Primary breast cancer gene expression data in METABRIC

Gene expression data from METABRIC^15^ are obtained from the public domain training data. Normal samples are excluded from analysis, and subtypes are defined using the PAM50 class available in the clinical data. Supplementary Table 2 summarizes the clinical attributes of the 897 primary tumors by these subtypes. We compute survival times from the difference between diagnosis and follow-up dates. Patients are considered to have an event if they died of their disease, as indicated with the label “d-d.s” in the “last follow up status” variable.

We link METABRIC gene identifiers of the genes of interest in Fig. 1. In each case, we select probes indicated as having “Perfect” evidence in the annotation. We select the probe that with the lowest p-value for differential expression between subtypes for genes with multiple probes based upon the differential expression analysis described below, listed in Table 2.

### Differential expression analysis

For both TCGA and METABRIC, one-sided t-tests are applied to each gene to compare expression of samples in each subtype to samples from all other subtypes. P-values are adjusted using the Benjamini-Hotchberg procedure to account for multiple hypothesis testing. In the case of METABRIC, differential expression and survival statistics are reported for the probe that has the lowest p-value in any subtype relative to the other subtypes for each gene. All analyses are performed in R, version 3.1.1.

### Survival analysis

Due to the relatively limited follow-up time in TCGA, survival analyses are performed only for METABRIC data. The function “survdiff” in the CRAN package survival (2.37.7) applies the *G-rho* family of tests to compare survival curves for samples with high expression to samples with low expression to each gene in Fig. 1 that is also measured in METABRIC. We distinguish samples as having high or low expression of a gene relative to the distribution of expression values in the subtype that has lowest average expression of that gene. Specifically, a sample is defined as having high expression of a gene if its expression is at least one standard deviation above its mean expression in the subtype with lowest average expression. We do not perform analysis on combinations of genes and subtypes that have fewer than 10 samples with high or low expression.

## Additional Information

**How to cite this article**: Fertig, E. J. *et al.* Analysis of gene expression of secreted factors associated with breast cancer metastases in breast cancer subtypes. *Sci. Rep.* **5**, 12133; doi: 10.1038/srep12133 (2015).

## Supplementary Material

Supplementary Information

## Figures and Tables

**Table 1:** Pro-metastatic factors analyzed in this study.

Pro-metastatic factor	TCGA	METABRIC
IL6	IL6	ILMN_1699651
GP130	IL6ST	ILMN_1849013
GMCSF	CSF2	ILMN_1661861
GMRA	CSF2RA	ILMN_2376455
GRMB	CSF2RB	ILMN_1798475
CCL5	CCL5	ILMN_2098126
CCR5	CCR5	
VEGFA	VEGFA	
VEGFR2	KDR	ILMN_1686405
NRP1	NRP1	ILMN_1742547
VEGFC	VEGFC	ILMN_1701204
VEGFR3	FLT4	ILMN_2390427
NRP2	NRP2	ILMN_1787190

List of pro-metastatic factors considered in this study (Fig. 1). The TCGA column indicates gene aliases used to match the gene annotations in TCGA. Similarly, the METABRIC column indicates the single array probe used to obtain data for each gene in METABRIC, as described in the methods. A blank entry indicates that no measurements were available for that gene.

**Table 2:** Correlation between gene expression of pro-metastatic ligand and receptor pairs.

Ligand	Receptor	TCGA		METABRIC	
		R	p-value	R	p-value
IL6	GP130 (IL6ST)	−0.18	_3 × 10^–6^_	_−0.20_	_3 × 10^−9^_
CSF2 (GMCSF)	GMRA (CSF2RA)	0.34	_<2 × 10^−16^_	_0.23_	_8 × 10^−12^_
	GMRB (CSF2RB)	0.42	_<2 × 10^−16^_	_0.12_	_4 × 10^−4^_
CCL5	CCR5	0.86	_<2 × 10^−16^_		
VEGFA	VEGFR2 (KDR)	0.11	_6 × 10^−3^_		
	NRP1	0.06	0.13		
VEGFC	VEGFR3 (FLT4)	0.49	_<2 × 10^−16^_	_0.05_	_0.1_
	NRP2	0.42	_<2 × 10^−16^_	_0.16_	_3 × 10^−6^_

Spearman correlation coefficient (R) and corresponding p-value for mRNA expression of a ligand with its associated receptor(s) (Fig. 1) from RNA sequencing data from primary breast tumors in TCGA and microarray gene expression data from primary tumors in METABRIC. Gene names are consistent with Fig. 1, with aliases used in TCGA or METABRIC in parentheses.
