# Supplementary material for: Analysis of gene expression of secreted factors associated with breast cancer metastases in breast cancer subtypes
Source: Sci Rep. 2015 Jul 15;5:12133. doi: 10.1038/srep12133 (PMC4648401; doi:10.1038/srep12133)
Supplement: Supplementary Information [file srep12133-s1.pdf]

Analysis of gene expression of secreted factors associated with breast cancer metastases in breast cancer subtypes

Elana J. Fertig[1,*], Esak Lee[2,*], Niranjan B. Pandey[2], Aleksander S. Popel[1,2,**]

[1]Department of Oncology, Sidney Kimmel Comprehensive Cancer Center, Johns Hopkins University, Baltimore, MD, USA

[2]Department of Biomedical Engineering, Johns Hopkins University School of Medicine, Baltimore, MD, USA

*These authors contributed equally.

**Corresponding author
Department of Biomedical Engineering
Johns Hopkins University School of Medicine
611 Traylor Research Building, 720 Rutland Avenue
Baltimore, MD 21205, United States
Tel: 410-955-6419
Fax: 410-614-8796
Email: apopel@jhu.edu

Supplemental Table 1 Summary of characteristics of primary breast cancer tumor samples from TCGA that have RNA-sequencing data. Samples are grouped by subtype, which is defined by measurements of ER, PR, and HER2 receptor status.

	**Basal**	**HER2**	**Luminal A**	**Luminal B**
n	100	34	391	113
Age				
Min	29	34	28	29
Median	54	56	59	61
Max	90	80	90	90
Menopausal state				
Pre	29	7	90	23
Peri	4	2	14	3
Post	61	23	263	77
Indeterminate	2	0	1	0
Stage				
I	15	1	73	13
II	63	23	218	68
III	18	9	91	29
IV	1	0	4	2
X	2	1	5	1

Supplemental Table 2 Summary of characteristics of primary breast cancer tumor samples from METABRIC. Samples are grouped by PAM50 subtype.

	**Basal**	**HER2**	**Luminal A**	**Luminal B**
n	114	79	451	253
Age				
Min	27	22	28	29
Median	51	55	62	64
Max	79	90	90	92
Menopausal state				
Pre	53	24	99	35
Post	60	53	345	217
ER Status				
Negative	103	57	8	5
Positive	11	22	443	248
Stage				
0	58	48	229	117
1	17	10	93	50
2	32	12	116	73
3	7	9	12	10
4	0	0	1	3

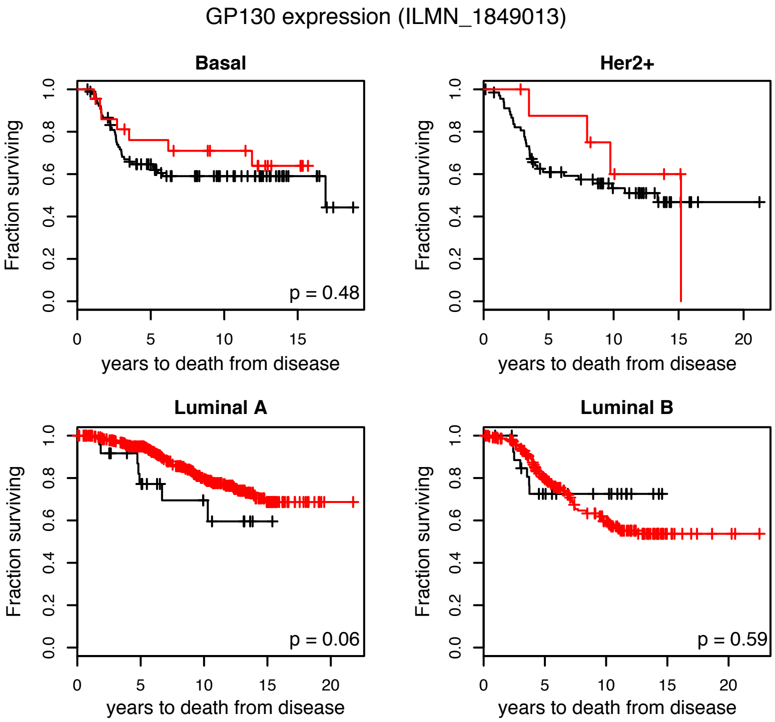

Supplemental Figure 3. Fraction of patients in each Pam50 subtype surviving during the METABRIC follow-up period. Red lines plot survival in samples with greater than one standard deviation above the mean expression of *GP130* in the subtype with lowest average expression (Her2+), with black lines plotting the remaining samples. Reported p-values test for differences between the survival curves high expressing (red) and low expressing (black) groups of samples when there are sufficient samples in each group to test.

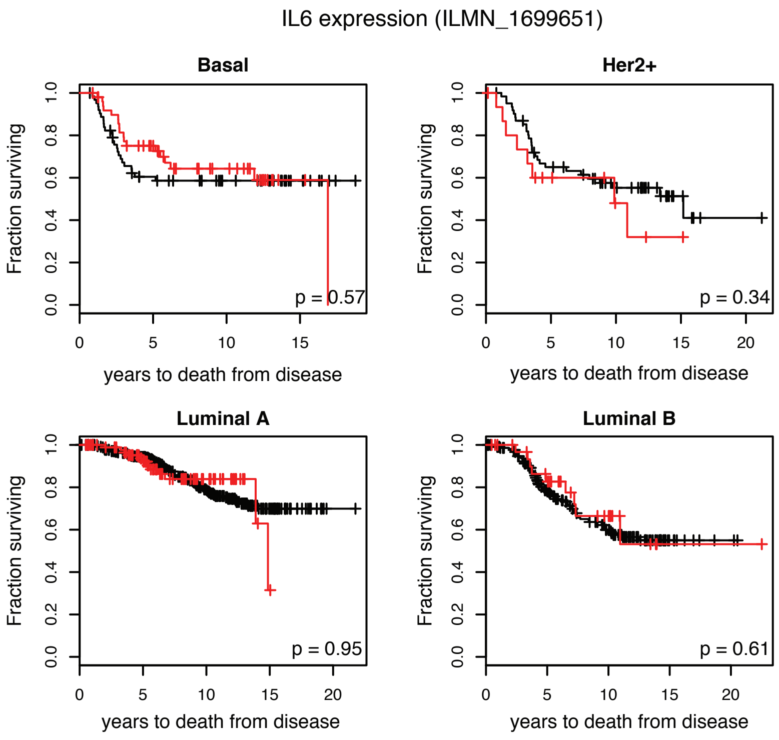

Supplemental Figure 4. Fraction of patients in each Pam50 subtype surviving during the METABRIC follow-up period. Red lines plot survival in samples with greater than one standard deviation above the mean expression of *IL6* in the subtype with lowest average expression (Luminal B), with black lines plotting the remaining samples. Reported p-values test for differences between the survival curves high expressing (red) and low expressing (black) groups of samples when there are sufficient samples in each group to test.

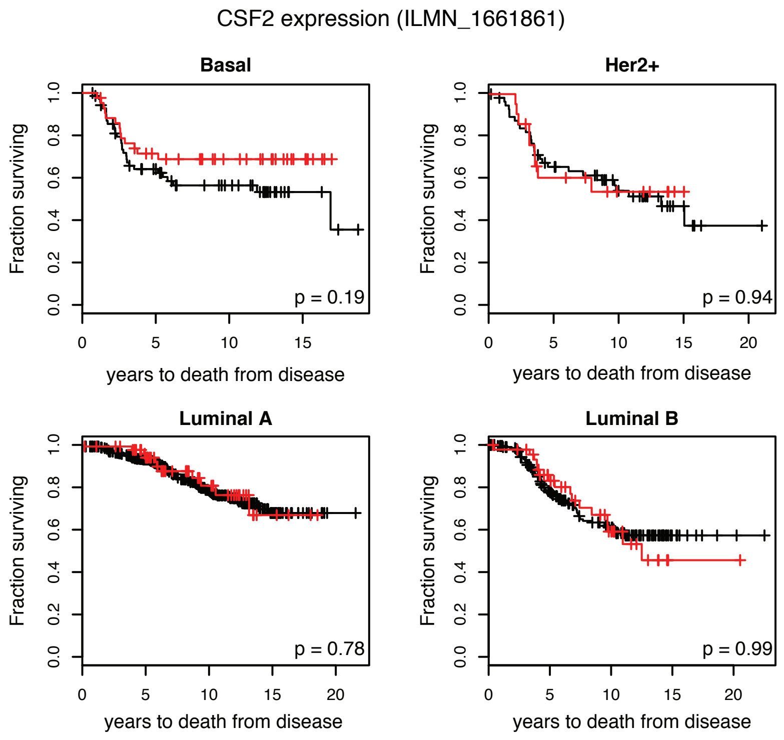

Supplemental Figure 5. Fraction of patients in each Pam50 subtype surviving during the METABRIC follow-up period. Red lines plot survival in samples with greater than one standard deviation above the mean expression of *CSF2* in the subtype with lowest average expression (Luminal B), with black lines plotting the remaining samples. Reported p-values test for differences between the survival curves high expressing (red) and low expressing (black) groups of samples when there are sufficient samples in each group to test.

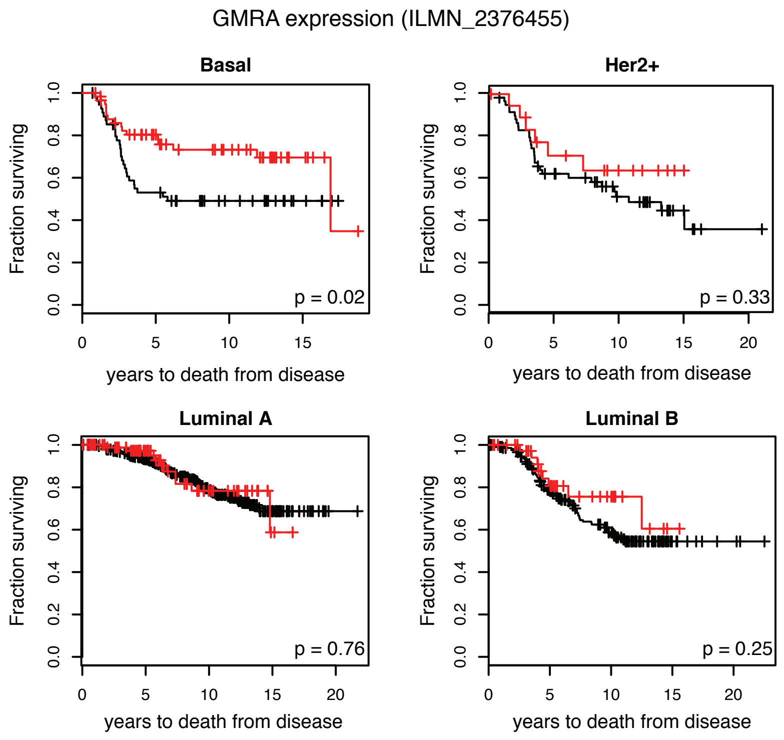

Supplemental Figure 6. Fraction of patients in each Pam50 subtype surviving during the METABRIC follow-up period. Red lines plot survival in samples with greater than one standard deviation above the mean expression of *GMRA* in the subtype with lowest average expression (Luminal B), with black lines plotting the remaining samples. Reported p-values test for differences between the survival curves high expressing (red) and low expressing (black) groups of samples when there are sufficient samples in each group to test.

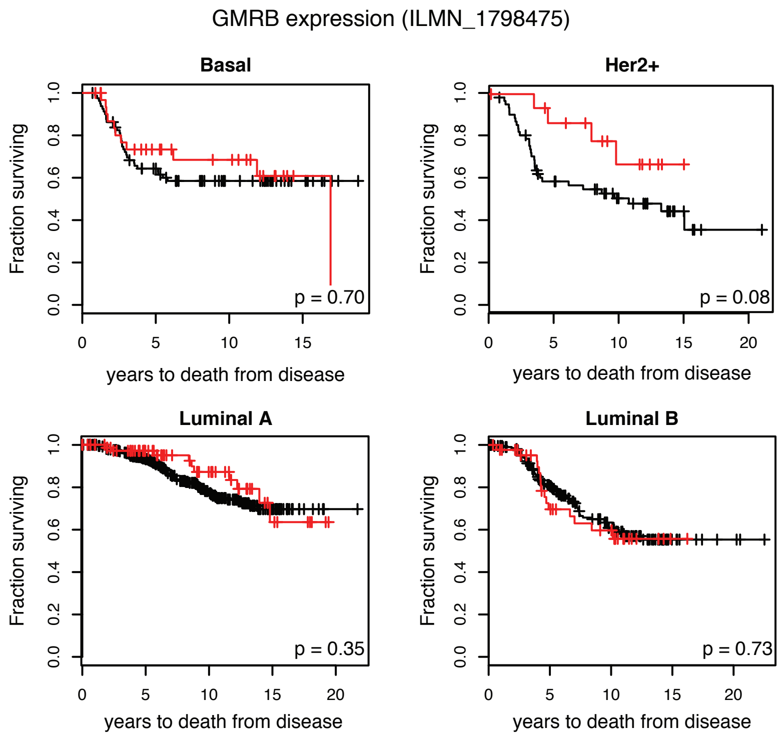

Supplemental Figure 7. Fraction of patients in each Pam50 subtype surviving during the METABRIC follow-up period. Red lines plot survival in samples with greater than one standard deviation above the mean expression of *GMRB* in the subtype with lowest average expression (Luminal B), with black lines plotting the remaining samples. Reported p-values test for differences between the survival curves high expressing (red) and low expressing (black) groups of samples when there are sufficient samples in each group to test.

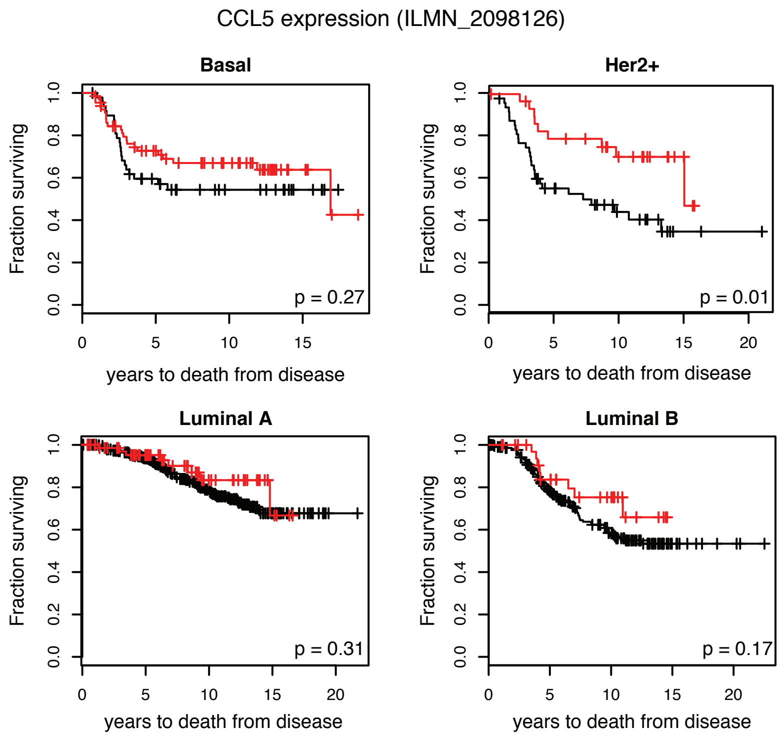

Supplemental Figure 8. Fraction of patients in each Pam50 subtype surviving during the METABRIC follow-up period. Red lines plot survival in samples with greater than one standard deviation above the mean expression of *CCL5* in the subtype with lowest average expression (Luminal B), with black lines plotting the remaining samples. Reported p-values test for differences between the survival curves high expressing (red) and low expressing (black) groups of samples when there are sufficient samples in each group to test.

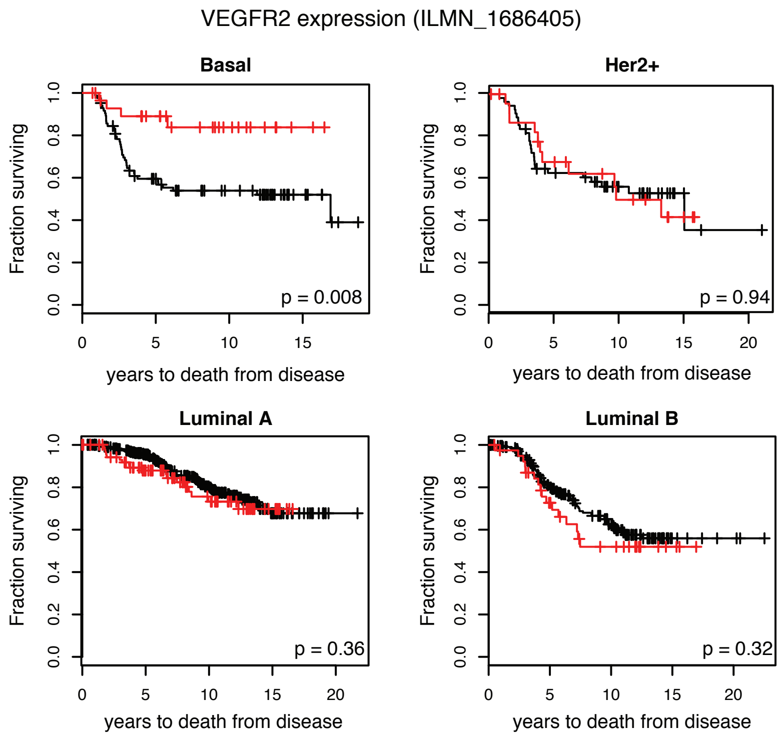

Supplemental Figure 9. Fraction of patients in each Pam50 subtype surviving during the METABRIC follow-up period. Red lines plot survival in samples with greater than one standard deviation above the mean expression of *VEGFR2* in the subtype with lowest average expression (Luminal B), with black lines plotting the remaining samples. Reported p-values test for differences between the survival curves high expressing (red) and low expressing (black) groups of samples when there are sufficient samples in each group to test.

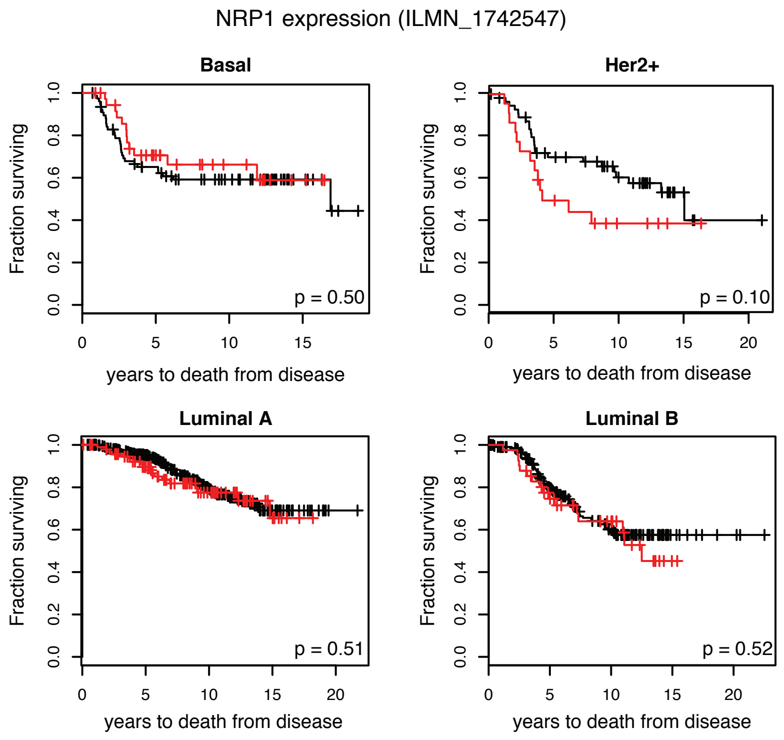

Supplemental Figure 10. Fraction of patients in each Pam50 subtype surviving during the METABRIC follow-up period. Red lines plot survival in samples with greater than one standard deviation above the mean expression of *NRP1* in the subtype with lowest average expression (Luminal B), with black lines plotting the remaining samples. Reported p-values test for differences between the survival curves high expressing (red) and low expressing (black) groups of samples when there are sufficient samples in each group to test.

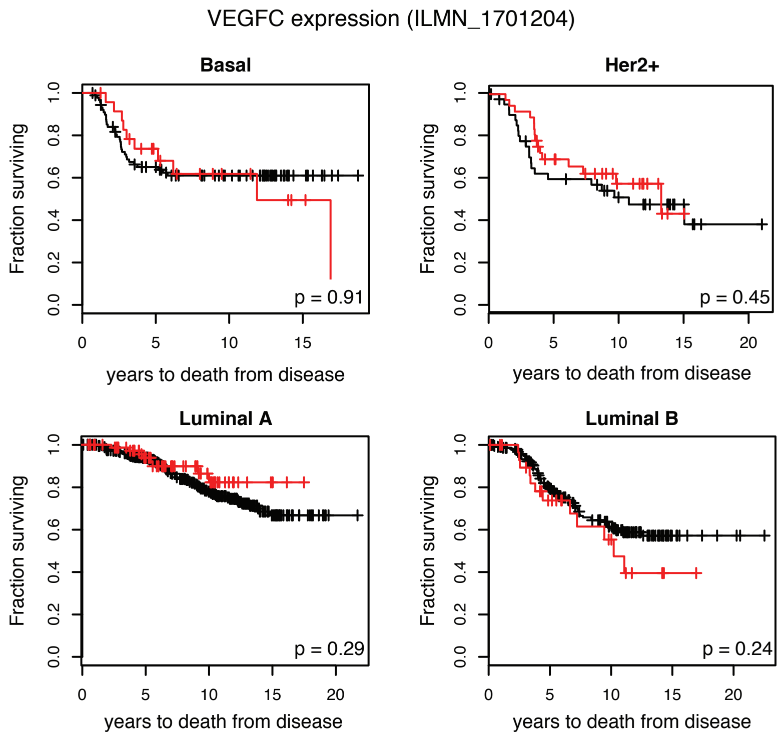

Supplemental Figure 11. Fraction of patients in each Pam50 subtype surviving during the METABRIC follow-up period. Red lines plot survival in samples with greater than one standard deviation above the mean expression of *VEGFC* in the subtype with lowest average expression (Luminal B), with black lines plotting the remaining samples. Reported p-values test for differences between the survival curves high expressing (red) and low expressing (black) groups of samples when there are sufficient samples in each group to test.

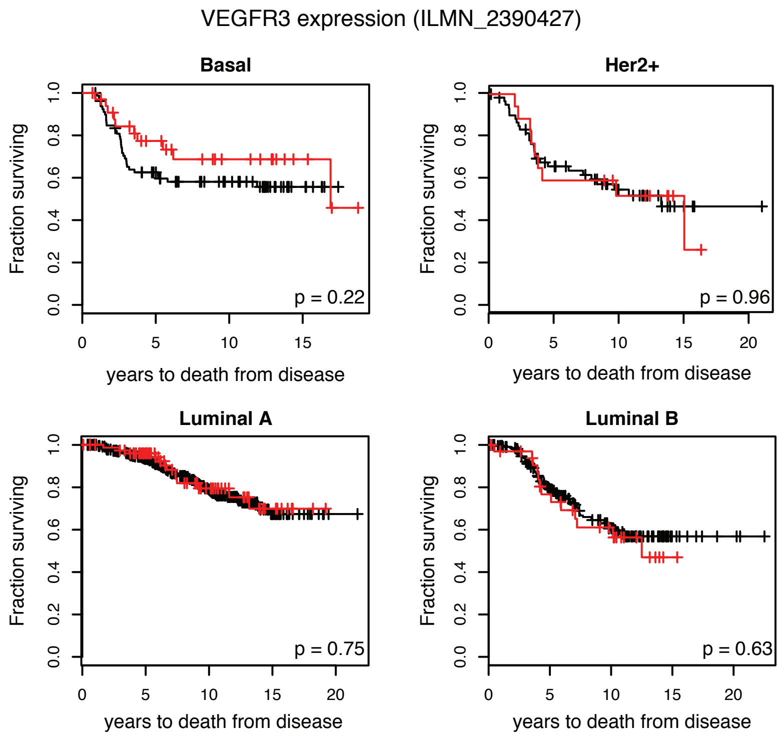

Supplemental Figure 12. Fraction of patients in each Pam50 subtype surviving during the METABRIC follow-up period. Red lines plot survival in samples with greater than one standard deviation above the mean expression of *VEGFR3* in the subtype with lowest average expression (Luminal B), with black lines plotting the remaining samples. Reported p-values test for differences between the survival curves high expressing (red) and low expressing (black) groups of samples when there are sufficient samples in each group to test.

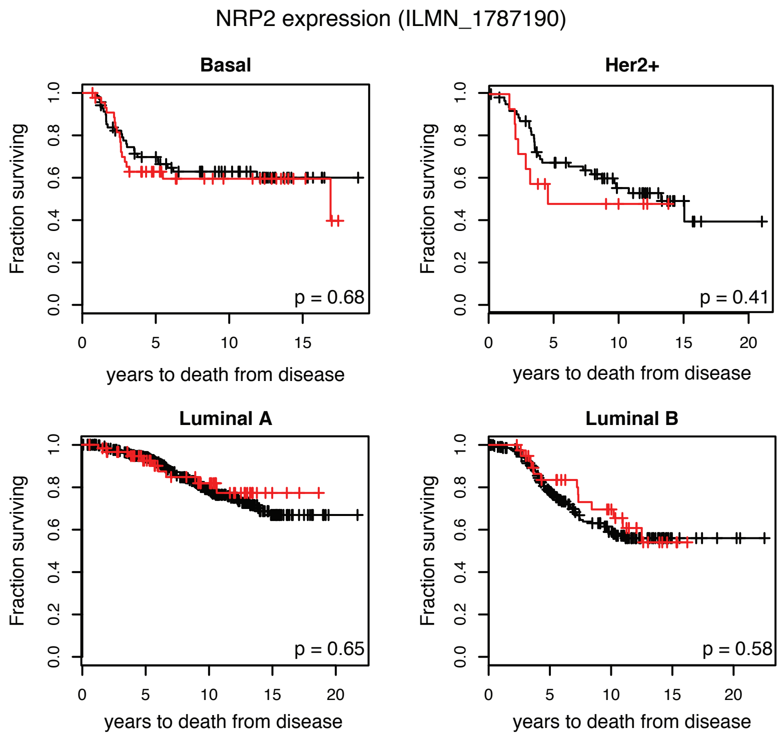

Supplemental Figure 13. Fraction of patients in each Pam50 subtype surviving during the METABRIC follow-up period. Red lines plot survival in samples with greater than one standard deviation above the mean expression of *NRP2* in the subtype with lowest average expression (Luminal B), with black lines plotting the remaining samples. Reported p-values test for differences between the survival curves high expressing (red) and low expressing (black) groups of samples when there are sufficient samples in each group to test.
